# The quest for good explanations

**DOI:** 10.1371/journal.ppat.1006818

**Published:** 2018-03-08

**Authors:** Benhur Lee

**Affiliations:** Department of Microbiology, Icahn School of Medicine at Mount Sinai, New York, New York, United States of America; The Fox Chase Cancer Center, UNITED STATES

"…all progress, both theoretical and practical, has resulted from a single human activity: the quest for what I call good explanations."

—David Deutsch, *The Beginning of Infinity* (2012)

I often tell people that if I didn’t have to work for a living, I would still be doing what I’m doing. It is an unbelievable privilege to be an academic scientist, paid to work on your passion, to follow up on your curiosity, to inspire and be inspired by generations of graduate students and post-docs that pass through your lab. So how did I end up being a scientist, doing what I love for a living?

I grew up in Singapore in the 1970s and early 1980s. I was educated in a school system that encouraged the rote memorization of facts. Science, as it was taught in my high school, didn’t allow room for questions that might lead to independent thinking. Facts had to be learned only well enough to pass examinations. If one did well in science, one went to medical school. One only saw scientists in movies, never in real life.

When I was 17, I went off to college in America, and my eyes were opened. I remember writing in wonder to my friends back home to tell them about a time in freshman chemistry class when one student asked a question to which the professor replied, “I’m still working on that”. This is what I wanted to do! I want to be part of a profession that generated knowledge with which to explain things better.

But my path to becoming a professional scientist and a virologist is best described as circuitous. During college, I worked in an exobiology lab at NASA’s Ames Research Center studying lipid biosynthesis in certain cyanobacteria species. I was fascinated that we could gain insights into Earth’s ancient atmosphere by studying “fossilized” metabolic pathways that could be reactivated only in the absence of oxygen. I was amazed we could tease out secrets that had been hidden for more than a billion years. I wanted to be a member of a profession capable of such elegant detective work. I thought being a physician scientist offered me a way to satisfy my love for science and medicine. Unfortunately, federally funded MD/PhD programs were not open to foreign students, so I went to the only medical school (Yale) that required an original thesis for graduation.

I ended up taking an extra year off to do research, working on the molecular cell biology of small nucleolar ribonucleoproteins, which only fortified my desire to become a scientist. After medical school, I headed off for a research residency at the University of Pennsylvania, where I did my postdoctoral research with Bob Doms. The coreceptors for HIV entry had just been discovered when I joined Bob’s lab. For a while, it seemed like coreceptor-mediated entry had an explanatory role in almost every aspect of HIV pathogenesis. Doing science became addictive. Figuring out how the virus evolved to use different coreceptors was endlessly fascinating.

The continuing search for good explanations has powered my subsequent career as an independent scientist. From HIV to paramyxoviruses, some might describe my research as being peripatetic. My guiding principle, however, is the motto that “viruses are the best cell biologists.” How viruses enter, replicate, and exit their host cells is an evolutionary record of the most successful cell biological strategies that have survived eons of culling by natural selection. By studying how viruses co-opt basic cell biological processes to complete their life cycle, we aim not only to enhance our understanding of cell biology on a molecular level but also reveal targets for therapeutic interventions against a broader spectrum of viral pathogens.

My transition from studying HIV to paramyxoviruses was spurred by our finding the main receptor for henipavirus entry (ephrinB2). Henipaviruses were then just being recognized as a group of pathogenic zoonotic paramyxoviruses that cause fatal infections in humans, livestock, and susceptible animals. EphrinB2 is a highly conserved receptor tyrosine kinase. Its conservation explained why henipaviruses were able to infect such a broad range of animal hosts. Its expression in specific tissues and cell types, such as neurons and endothelial cells that line the smallest of blood vessels, explained how the virus was able to spread systemically and ultimately cause the fatal encephalitis that is a hallmark of henipaviral disease. We subsequently identified ephrinB3 as an alternate receptor. Although ephrinB3 is not expressed on endothelial cells, it is expressed in certain areas of the brain where ephrinB2 is not. We hypothesized that ephrinB3-mediated entry contributes significantly to the brain pathology that is the ultimate cause of death. The discovery of novel henipaviruses in recent years that do not use ephrinB3 now allows us to test this hypothesis. Of course, the alarming number of novel henipaviruses also underscores the risk of global emergence of these zoonotic viruses. The search for good explanations continues as we seek to define the pathogenic potential of these novel viruses.

After several years working on the intricacies of how viruses get into cells, I started to wonder about how viruses get out. The paramyxovirus matrix (M) protein is critical to viral assembly and budding, but little was known about how this occurred. Much to our surprise, we found canonical nuclear localization and nuclear export sequences in many paramyxovirus M proteins. Our initial studies revealed that during early infection, the Nipah virus M protein is first targeted to the nucleus prior to ubiquitination and subsequent localization to the plasma membrane. This ubiquitin-regulated nuclear–cytoplasmic trafficking of M is critical for its ability to mediate viral assembly and budding at the plasma membrane. It is not only conserved in other henipaviruses, but viruses from several paramyxovirus genera. These findings have begun to shed light on the mechanistic basis for a long-standing and puzzling observation regarding the nuclear transit of some paramyxovirus M proteins, as paramyxoviruses replicate entirely in the cytoplasm. Trafficking of M proteins through the nucleus, a subcellular compartment incompatible with their known function (budding at the plasma membrane), hints at nonstructural roles for M. In ongoing collaborations, we have now identified the matrix interactomes from paramyxoviruses across the various genera. A treasure trove of biologically interesting interactions awaits good explanations!

**Image 1:**
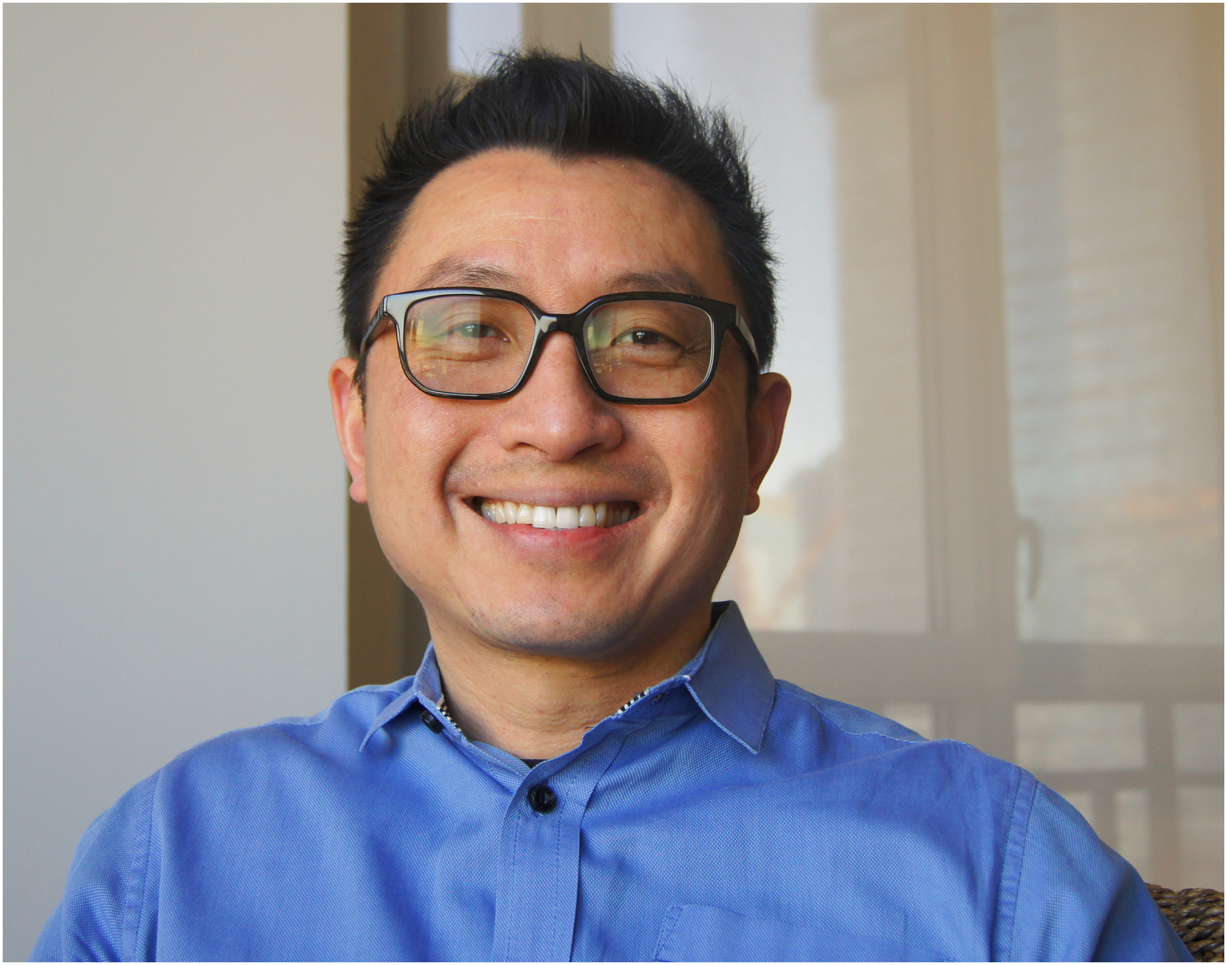
Benhur Lee.

